# Anticancer Activity of Ganoderic Acid DM: Current Status and Future Perspective

**DOI:** 10.4172/2155-9899.1000535

**Published:** 2017-12-12

**Authors:** John Matthew Bryant, Mollie Bouchard, Azizul Haque

**Affiliations:** Department of Microbiology and Immunology, and Hollings Cancer Center, Medical University of South Carolina, USA

**Keywords:** *Ganoderma lucidum*, Ganoderic acid-DM (GA-DM), Cell death, T cells, Anticancer

## Abstract

*Ganoderma lucidum* is a mushroom that has a long history of medicinal use in the Far East countries as this mushroom is revered for its supposed miracle cures and life improving properties. Recently, this mushroom has come under scientific scrutiny to examine the possibility of finding biologically active compounds that may have an impact on human physiology. The main category of biologically active compounds produced in the *G. lucidum*, are the triterpenoids, which are known as Ganoderic Acids. In this review, we discuss one Ganoderic Acid in particular known as Ganoderic Acid-DM (GA-DM) that is extracted from the *Ganoderma lucidum* mushroom. We will discuss GA-DM as a potential therapeutic candidate for treating a number of diseases yet will focus on the potential to be used as an alternative or supplemental therapeutic agent in regards to various cancer types. The urge for this promising therapeutic agent is that GA-DM is capable of inducing cell death in cancer cells while exhibiting minimal toxicity to normal bystander cells. Furthermore, this review will look at GA-DM’s ability to stimulate an immune response in the tumor environment to potentially provide long-term protection from the malignant tumors. We will also discuss the known routes of administration of GA-DM and pose the advantages and disadvantages of each route in a comparative manner. Finally, we will cover current status of the roles GA-DM may have as a therapeutic agent in respect to different cancer types as wells as discuss about its future perspective as a therapeutic candidate in other diseases as well.

## Introduction

Mushrooms have a long history of medicinal use predominately by Far East Countries dating back more than four thousand years [1-5]. A fair number of mushrooms used in Asian countries are revered for their supposedly miracle cures and general life improving properties [5]. Numerous mushrooms are cultivated and used as herbal medicine despite any empirical evidence for benefit except for anecdotal evidence or conclusions drawn from animal models experiments [5-7]. One of the more popular medicinal mushrooms is *Ganoderma lucidum* which comes from the shiny appearance of its fruiting body [1,8]. The more common names of *Ganoderma lucidum* however, come from its purported health benefits rather than its physical appearance. In China and Korea, the *Ganoderma lucidum* is known as the lingzhi and is regarded as the “herb of spiritual potency” [6]. In Japan, *Ganoderma lucidum* is called reishi or mannentake and is regarded as the ten thousand year mushroom [1]. This mushroom is reported in Asian populations as having miraculous curative properties whose medical claims can be attributed to a well-respected pharmacopeia from the Qin dynasty (221-206 B.C.) called Shen Nong Ben Cao Jing or The Divine Farmer’s Materia Medica [5]. This medical practice of consuming *Ganoderma lucidum* caused this mushroom to have a widespread effect on the culture so as to appear in numerous pieces of artwork and literature beginning in the Yuan Dynasty (1280-1368 A.D.) [1,5]. The consumption of *Ganoderma lucidum* by Far East countries for medical purposes takes on different methods of ingesting the mushroom. While this mushroom is not toxic, it is very difficult to ingest. The mushroom is very tough so that it is not edible in its raw state and that the compounds in it are incredibly bitter [1,5,6]. The method of ingestion lies in turning the mushroom into a powder and modifying the route to ingest the powder. *Ganoderma lucidum* is manufactured into a number of commercial products such as powders, supplements, and tea [5,6]. In herbal medicine practices in the Far East, *Ganoderma lucidum* is prescribed in different ways [1,5]. The methods range from injecting a solution of powdered spores to drinking a soup, syrup, tea, capsule, tincture, or bolus with the mushroom powder in the concoction [1]. While the practice of herbal medicine is accepted in the Far East, the same practice is met with great skepticism in the West. However, in recent years a plethora of research investigating the clinical benefits of certain botanicals have yielded intriguing results by discovering a slew of biologically active compounds that may eventually lead to the development of more pharmaceuticals to treat various ailments. The examination of botanicals that have a medical practice history spanning thousands of years in the search for new pharmaceuticals will probably yield some advantages to elucidating new drugs that can be used in conventional medical practices. As an example, recent research on *Ganoderma lucidum* has illuminated some interesting compounds that may have clinical significance.

*Ganoderma lucidum* contains a wide variety of bioactive compounds, such as, terpenoids, steroids, phenols, and nucleotides and their derivatives, glycoproteins, and polysaccharides [1,2,5,6]. The biologically active molecules that may have a particular interest in the clinical setting are the terpenes and more specifically, Ganoderic Acids (GAs). Terpenes are a class of compounds produced by the *Ganoderma lucidum* which are carbon structures composed of one or more isoprene C5 units [6]. GAs are classified as Triterpenes, a subtype of triterpenoids, as they are composed of six isoprene units [5,9]. In general, triterpenoids have molecular weights ranging from 400 to 600 kDa and their chemical structure is complex and highly oxidized [5,6]. GAs are composed of four cyclic and two linear isoprene units. There are over 140 different GAs that have been identified from *Ganoderma lucidum*. These GAs are identified by the different R-groups. These triterpenes are of interest in research as a number of these compounds have been found to target cellular processes such as apoptosis, cell cycle regulation and angiogenesis through direct interaction with molecular targets [1,5,6,10].

The focus of this review article will be on one of the triterpenoids produced by the *Ganoderma lucidum* mushroom, which is Ganoderic Acid-DM (GA-DM). GA-DM comes from the same *Ganoderma lucidum* that produces the other GAs as well. The main source of GA-DM is through the cultivation of *Ganoderma lucidum* for extraction of the triterpenes present in the fungus [5,9]. There is an issue with this as through the artificial cultivation of *Ganoderma lucidum*, a low yield of GAs, specifically GA-DM, is produced [11]. It usually takes several months to cultivate the fruiting body of the fungus and is incredibly difficult to control the amount of the active compounds produced by the fungus during cultivation [5,11]. The research on the enhancement of GA production in this fungus has divided into two main branches. The first is focusing on the growing environment and examining the effects of GA production. Researchers in this field have discovered that the production of GAs can be manipulated by a number of different factors in the environment [5,6,11]. A method of cultivation is through submerged fermentation of the fungus. This process is used as an alternative method for the efficient production of GAs [5]. Researchers have found that by manipulating the growing conditions such as the medium, oxygen supply, and the pH, the production of GAs in the mycelium can be enhanced. The second branch of enhancing GAs production is through regulating gene expression levels for those genes specifically involved in the biosynthesis of these GAs. There is a separate method of increasing the levels of specific GAs, such as GA-DM, by using additional chemical conversion processes to convert the analogue impurities to the desired compound.

In *Ganoderma lucidum*, the chemical structure of the triterpenes is based on lanostane, which is a metabolite of lanosterol, the biosynthesis of which is based on cyclization of squalene [3,6,12,13]. Once the fruiting body of the *Ganoderma lucidum* has developed, they are collected and processed to extract the triterpenes from the fungus. Extraction of triterpenes is usually done by means of methanol, ethanol, acetone, chloroform, ether, or a mixture of these solvents. The extracts can be further purified by various separation methods, including normal and reverse-phase HPLC. The triterpenoid of interest in this review is GA-DM.

## Anticancer Activity of GA-DM

GA-DM has taken great interest in the research of treating diseases. The main area of research to examine the utilization of GA-DM is in cancer therapies. So far there have been studies published on the role of GA-DM in treating prostate cancer, melanoma, breast cancer, meningioma [3,14-17], and a study ongoing in our laboratory to look at a possible use in treating lymphoma. The interest in GA-DM is that it may offer an alternative treatment in regards to cancer therapy drugs. The urge to find alternatives in terms of treating prostate cancer is that the conventional therapies while effective at clearing the cancer in its early stage, are often ineffective at treating prostate cancer that is in the late metastatic stages [16,18,19]. The current problem with treating melanoma is that the standard therapies are often ineffective at making a substantial impact on the survival of the patients [15,20]. The urge to find an alternative treatment for breast cancer is that the efficacy of the current chemotherapeutics is limited by intrinsic and acquired therapeutic resistance, and thus it is urgent to find an alternative approach to combat the disease [3,21]. The urge to find an alternative treatment for meningioma is that there is a significant problem in establishing a beneficial impact on recurrent meningiomas with current chemotherapies [22]. The urge for an alternative treatment for lymphoma, specifically Diffuse Large B-cell Lymphoma (DLBCL) is that depending on the subtype of DLBCL diagnosed, the patients experience significantly different survival rates following chemotherapy [7,23]. Studies have been conducted using medicinal mushrooms while screening for bioactive compounds that may have the desired effect to treat these cancers and the papers published indicate that GA-DM is a potential candidate for acting as an alternative or supplemental therapy in regards to the previously mentioned cancer types.

The research on medicinal mushrooms has led to the discovery of a number of different biologically active compounds. The *Ganoderma lucidum* has eluted a number of compounds that have a direct interaction with target molecules in certain cellular processing pathways. A compound of interest is the triterpenoid called GA-DM which has been tested in a number of studies in an attempt to find an alternative or supplemental therapeutic drug for different cancers. While there are studies dealing with GA-DM and its use in treating other disease, recent work has been shifted to cancer therapy which will be the main focus of this review. The studies published give rise to future prospects of utilizing GA-DM in cancer therapy as an alternative and or a supplemental treatment for combating advanced carcinomas. Table 1 shows an overview of current status of GA-DM as a therapeutic agent in disease treatment.

## GA-DM as an Alternative Chemotherapeutic Agent

**Figure 1:**
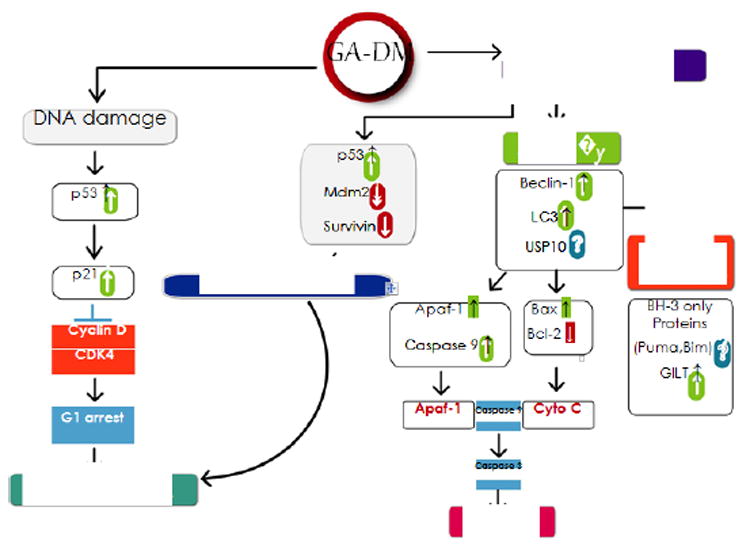
Proposed mechanisms by which GA-DM may exhibit anticancer activity. GA-DM may induce a crosstalk between autophagy and apoptosis, leading to an enhanced immune recognition of tumor. GA-DM treatment induces cell cycle arrest (G1 phase), downregulates survival proteins (bcl-2, Mcl-1, survivin, etc), upregulates apoptosis related proteins (Bax, Apaf-1), activates effector caspases (caspase 3), and may ultimately induce tumor cell death regardless of p53 status. GA-DM treatment may also disrupt p53/mdm^2^ and upregulate autophagic (Beclin-1, LC3) and immunomodulatory components.

There are three main approaches to treat cancer: surgery, radiation, and chemotherapy [24-26]. Chemotherapy involves the use of drugs that either kill cancer cells or interfere with the ability of cancer cells to proliferate. This form of therapy is effective for treating metastatic cancers due to the fact that the drugs can travel through the bloodstream to reach cancer cells wherever they may have spread. Unfortunately, this adds certain toxic side effects as the most anticancer drugs are toxic to dividing cells in general. GA-DM is capable of inducing cell death in various types of cancer cells while not displaying the same cytotoxicity to bystander cells [4,15,16]. There are three main pathways that can lead to cell death: apoptosis, autophagy, and necrosis [22,27,28]. Apoptosis is the main cell death pathway that is achieved through cellular processes and generally poses some beneficial effects to the organism [15,28]. Necrosis is another cell death pathway yet does not follow the apoptotic pathway. Necrosis uses various receptors that result in the loss of the cell membrane stability causing the release of cellular components typically producing negative impacts on the organism [23,28,29]. Autophagy is typically associated with cell survival yet can also play a role in cell death [15,29-31]. It is the cell’s own processes for protein degradation as a sort of repair or recycling mechanism. If this pathway were to be substantially upregulated, then the autophagic molecules can degrade cellular proteins to such a degree that the cell dies [28]. Based on research currently available, GA-DM is capable of inducing apoptosis and autophagy in various cancer cell types yet does not trigger necrosis. As shown in Figure 1 there is a hypothetical model in regards to how GA-DM may convey a therapeutic effect on tumor growth. The figure hypothesizes the therapeutic effects in respect to individual molecular targets and the cellular processes associated with those particular proteins. The importance of cell death induced by GA-DM is that it works *via* apoptotic and autophagic pathways, and thus may offer an alternative to conventional chemotherapeutics. Furthermore, there is much anticipation for GA-DM to help produce long-term effects to cancer patients by playing a role in stimulating the immune system against various cancer types.

## GA-DM as an Immune Stimulatory Agent in Cancer Therapy

Studies suggest that GA-DM is capable of inducing cell death yet stimulating the immune system. We have shown that GA-DM induces cell death through apoptosis and autophagy which enhances tumor antigen (Ag) presentation to CD4+T cells [15]. GA-DM upregulates an autophagic protein, Beclin-1, which binds to the survival protein Bcl-2, triggering apoptosis mediated by the activation of caspase 3 [15]. Our study suggests that autophagic processes may enhance Ag processing and presentation *via* HLA molecules which is crucial for the development of immunity against malignant tumors. This study also showed a significant upregulation of HLA class II molecules as well as lysosomal LAMP-2 proteins when treated with GA-DM. This correlated with autophagic processes conveyed by Beclin-1 expression in the melanoma cells. Our laboratory also analyzed the HLA class II components by western blotting, where a significant increase in HLA-DR and HLA-DM proteins with a differential Ii expression was detected, indicating activation of Ag processing machinery in the GA-DM treated melanoma cells [15]. Our study also looked at cell-mediated immune activation and recognition of GA-DM-treated melanoma *in vitro* by using whole HSA protein or HSA64 76K peptide as a model for the study of Ag processing and presentation to specific CD4+ T cells. Data obtained suggested that GA-DM treatment increases HLA class II Ag presentation and CD4+ T cell recognition of melanoma tumor cells.

## GA-DM in Cancer Chemoimmunotherapy

Chemoimmunotherapy is a combination of conventional chemotherapy and immunotherapy [32-34]. Chemotherapy uses different drugs to kill or slow the growth of cancer cells while immunotherapy uses treatments to stimulate or restore the ability of the immune system to fight cancer. A common chemoimmunotherapy treatment is CHOP combined with rituximab for B-cell non-Hodgkin lymphomas. CHOP therapy consists of Cyclophosphamide, Doxorubicin, Vincristine, and Prednisone [33-35]. Rituximab is a chimeric monoclonal antibody against the CD20 protein that is present on the surface of B cells [35,36]. Rituximab binds to this surface protein and destroys the B cells both healthy and malignant. This antibody is added to CHOP therapy to be classified at chemoimmunotherapy known as R-CHOP as the antibody has an impact on the immune system of the patient. GA-DM could be used in chemoimmunotherapy due to the fact that research indicates GA-DM’s ability to stimulate an immune response while inducing cell death in various types of cancer. It is capable of enhancing tumor Ag processing and presentation to CD4+ T cells which aids in the recognition of the tumor cells by the immune system and providing lasting immunity to malignant tumors [15]. Furthermore, research into treating various cancer types with GA-DM reveals that different methods of administration may present with a greater decrease in tumor burden and delayed disease progression for those suffering from malignancies.

## Routes of GA-DM Administration in Cancer Therapy

There are two main methods of GA-DM treatment administration in cancer therapy: (a) systemic administration and (b) nanoparticle mediated delivery. These routes of administration are different yet pose different advantages and disadvantages in terms of combating cancer progression. The systemic administration offers a broader area of coverage when introduced into the patient. However, GA-DM does not easily enter cells and so a greater concentration of this compound is required to establish a therapeutic effect. Unfortunately, this higher concentration typically leads to significant toxicity towards actively proliferating nonmalignant cells [4]. The use of GA-DM encapsulated nanoparticles reduces the required concentration of GA-DM significantly and focuses the treatment to a local area as opposed to systemic circulation if simply injected as the nanoparticles deliver the drug directly to the malignant tumor cells.

Preliminary studies from our laboratory indicate that there is a substantial change in dosage depending on the route of administration. These studies suggest that through systemic administration of GA-DM, a therapeutic effect can be observed when the concentration of GA-DM is around 20 μM [4], yet is in the nM range when using the nanoparticle route (unpublished data). The decrease in concentration with the nanoparticles suggests that there will be less cytotoxic effects to normal healthy cells. Furthermore, the nanoparticle method may help reduce metastasis as the drug can be delivered to an area that the malignant cells prefer to metastasize to.

## Diseases Involved in GA-DM Research

### Prostate cancer

Prostate cancer is the most commonly diagnosed cancer in men, and is the second most common cause of cancer-related deaths in the western world [16,37,38]. There is a growing interest in developing more effective treatments options due to the frequency and mortality associated with prostate cancer. Currently, when a patient is diagnosed with prostate cancer, the most common treatment options include prostatectomy, radiation, and chemotherapy [16,24,39]. While these treatment options are effective in treating most local forms of prostate cancer, many instances develop into castrate-resistant prostate cancer where alternative therapies are required. One of the most significant problems associated with prostate cancer is its ability to metastasize, particularly to bone [16,38,40]. Certain types of prostate cancer exploit a normal cellular process called osteoclastogenesis which leads to the formation of osteoclasts, large multinucleated cells that cause bone resorption and the metastatic prostate cancer colonizes this mineralized bone [16,19]. Immunotherapies to provide an effective long term treatment of advanced stage prostate cancer have been the goals of recent studies.

A potential candidate for chemoimmunotherapy in later-stage prostate cancer could be GA-DM as it induces cytotoxicity in both androgen dependent and independent prostate cancer cells [16]. It has been shown that GA-DM treatment inhibits both the activity of 5-α-reductase and the conversion of testosterone to Dihydrotestosterone (DHT). 5-α-reductase is crucial to androgen development as it reduces testosterone to its active form, dihydrotestosterone (DHT) [16,41]. The inhibition of DHT activity possibly occurs due to the conformational similarity in the structures of DHT and GA-DM. GA-DM competitively blocks the androgen receptors, preventing DHT binding and obstructing the normal DHT-mediated signaling pathway which results in cell survival. Furthermore, GA-DM has a potential effect of inhibiting osteoclastogenesis which is a major component of prostate cancer metastasis.

### Melanoma

Melanoma is the most aggressive form of skin cancer, responsible for the majority of skin cancer related deaths [15,42]. There is a search for a novel therapy that can destroy the tumors while simultaneously promoting an immune response against the metastatic melanoma tumors. A potential novel therapy for metastatic melanoma is GA-DM. We have also shown that GA-DM treatment induces apoptosis of melanoma cells [15]. In melanoma, GA-DM induces a cross-talk between autophagic and apoptotic cell death, as well as enhancing tumor Ag presentation *via* HLA class II. We have previously shown that gamma-interferon-inducible lysosomal thiol-reductase (GILT) enhances HLA class II Ag presentation in human melanoma cells [43].

Interestingly, GILT expression inhibits a tumorigenic molecule, paired box-3 (PAX-3) protein, in melanoma *via* the autophagy pathway [44]. It would be interesting to look at whether GA-DM alters GILT expression and immune recognition of melanoma for further investigation. Furthermore, GA-DM could initiate a possible cross-talk between autophagy and apoptosis, resulting in enhanced immune recognition of melanoma. This activation of the autophagic pathway or cross-talk has been shown to cause an upregulation of HLA class II proteins which enhanced tumor Ag presentation to CD4+ T cells [15]. *In vivo* experiments were conducted using B16 mouse melanoma model, and it was found that this enhanced Ag presentation led to greater T cell infiltration of the tumor tissue and clearance of melanoma when treated with GA-DM.

### Breast cancer

Breast cancer is the leading cause of cancer related death in women [3,45]. Treatment includes surgery, radiation, chemotherapy and hormonal therapy. The issue with treating breast cancer with chemotherapy is that the efficacy is limited due to therapeutic resistance in the cancer cells. GA-DM may be a candidate for treating breast cancer. Studies found that GA-DM can effectively inhibit cell proliferation and colony formation in breast cancer cells [3]. GA-DM has been shown to mediate G1 cell cycle arrest and decrease the protein level of CDK2, CDK6, cyclin D1, p-Rb and c-Myc [3]. Moreover, GA-DM induced DNA fragmentation and cleavage of PARP which are the characteristics of apoptosis and decrease the mitochondrial membrane potential in breast cancer cells. This finding is supported further in our study which showed that GA-DM elicits DNA damage in melanoma cells [15]. The slight upregulation of protein markers for DNA damage such as γ-H2AX when treated for 6 hours with GA-DM suggests that the G1 cell cycle arrest and apoptosis are partially mediated from the induced DNA damage [3].

Overall, this study showed that GA-DM could be a potential natural and alternative therapeutic agent for treating breast cancer.

### Osteoporosis

Osteoporosis is a common disease associated with high levels of bone resorption [46-48]. This ailment is typically seen in postmenopausal women. Conventional therapy involves hormone replacement therapy to prevent this bone loss, yet many women cannot tolerate the side effects of estrogen therapy or are uneasy about the possible risk of developing uterine and or breast cancer [46,49]. During the progression of the disease, osteoclasts play an important role in the bone resorption. Therefore, there is an interest to find a novel therapy that can target osteoclasts, and slow the progression of the disease.

GA-DM is capable of blocking osteoclastogenesis [19,46]. It has been shown that GA-DM suppresses the expression of cathepsin K and TRAP mRNA without affecting the mRNA level of GAPDH in a system of osteoclastogenesis using the RAW 264 cell-D clone [46]. These results confirmed the ability of GA-DM as a specific inhibitor of osteoclastogenesis. As cathepsin K is not only a marker of osteoclasts but is a critical protease for osteoclastic bone resorption, the inhibition of cathepsin K expression induced by GA-DM may be involved in the suppression of osteoclastic bone resorption seen in Ovx rats that are treated with GA-DM. Miyamoto et al. also examined the expression of a suspected transcription factor for osteoclastogenesis, NFATc1 [47]. They found that when the RAW 264 cells D-clone were treated with GA-DM, NFATc1 protein expression was significantly down-regulated and the trend was the same for c-Fos. Osteoclast differentiation is induced by macrophage-colony stimulating factor (M-CSF) and RANKL [46,50]. RANKL binds its cognate receptor RANK and induces expression of c-Fos [46,51]. The c-Fos induces NFATc1 expression and that c-Fos and NFATc1 cooperatively regulate osteoclastogenesis in response to RANKL stimulation. As described above, GA-DM inhibits osteoclastogenesis through the suppression of transcription factor NFATc1 [46]. Real-time PCR analysis showed that GA-DM down-regulates the expression of DC-STAMP mRNA, but not MFR. Because DC-STAMP is a target of RANKL stimulation, DC-STAMP expression is likely regulated by transcription factors c-Fos and NFATc1. Miyamoto et al. [47] also reported that GA-DM suppresses not only c-Fos induction but also NFATc1 upregulation by RANKL. This inhibition results in a strong inhibition of DC-STAMP expression during osteolastogenesis.

### Alzheimer’s disease

Alzheimer’s disease is a neurodegenerative condition characterized by conformational changes in proteins that cause intracellular aggregates and extracellular plagues [52,53]. Aβ (amyloid β-peptide) has a central role in Alzheimer’s disease where neuronal toxicity is linked to its extracellular and intracellular accumulation as oligomeric species. Searching for molecules that attenuate Aβ aggregation could uncover novel therapies for Alzheimer’s disease.

GA-DM may be a possible candidate for treating Alzheimer’s disease [52]. A study used T-Rex293 cells that produced Aβ42-EGFP and treated the culture media with GA-DM. The study found that GA-DM attenuates intracellular aggregation of Aβ42-EGFP. Specifically, the study determined that it does so indirectly. The investigators blocked the proteasome and autophagy clearance systems for the peptide and showed that GA-DM was not able to reduce the levels of aggregation suggesting that it may stimulate one or both clearance systems. However, when the autophagic route was blocked only, the GA-DM treatment was still effective yet the efficacy was impaired when the proteasome activity was inhibited which indicates that GA-DM enhances clearance of the peptide aggregates by proteasomal degradation and may not be through autophagy.

### Meningioma

Meningiomas are the second most common central nervous system tumor found in adults [22,54]. Meningiomas develop from the arachnoid cells of the meninges that cover the brain. Most meningiomas are benign, localized, non-aggressive, and non-invasive. Only the higher grade meningiomas are aggressive, malignant, and invasive which can cause multiple neurological and physiological complications [22,55-57]. Currently available medications, and radiation may work for a while but progression through these treatments are practically universal. There is an interest in developing a novel therapy for the aggressive and non-resectable meningiomas.

GA-DM may have the potential for treating these aggressive meningiomas, and seems to work *via* the Wnt5/GSK3β/β-catenin signaling pathway. Gsk3β has a major role in the Wnt/β-catenin signaling pathway. This study showed that GA-DM suppressed the expression of Wnt5α/β and β-catenin and enhanced the phosphorylation of GSK3β in IOMM-Lee and CH157MN cells in which the phosphorylation of Ser 9 is a marker for inactivation of GSK3β. This also suggests that GA-DM induced apoptosis is mediated by the Wnt5/GSK3β/β-catenin signaling. Furthermore, this study showed that GA-DM interrupts Wnt signaling by decreasing β-catenin activity, which in turn suppresses the expression of β-catenin target genes (c-myc, VEGF, and cyclin D1). GA-DM may also induce apoptosis *via* mitochondrial-dependent pathway as the study found caspase cascade activation and regulation of the Bcl-2 family proteins in IOMM-Lee and CH157MN cells [22]. GA-DM has also been shown to suppress anti-apoptotic proteins such as Akt, Bcl-XL, and Mcl-1 while it upregulates the expression of apoptotic protein Bax. These processes lead to the reduction of MMP and cytochrome C release.

The release of cytochrome c activates caspase cascade and PARP cleavage to induce apoptosis through the fragmentation of chromatin DNA.

## Conclusion

GA-DM may be a potential therapeutic candidate to treat an assortment of cancers as well as other diseases. This compound is capable of inducing apoptosis in cancer cells while exhibiting minimal toxicity to healthy cells. GA-DM is also capable of stimulating an immune response in the tumor environment to potentially provide long-term protection from the malignant tumors. The different routes of administration of GA-DM may lead to a number of different therapies based on the qualities each administration method possesses. While these studies provide great support for utilizing GA-DM as an alternative or supplemental therapy for various types of cancers, more research is required to better understand the full scope of molecular targets GA-DM acts on to develop a more efficacious therapy. The research currently available is promising and so further research should be greatly encouraged within the academic community to better understand how a natural mushroom product can be implemented into conventional medicine to treat malignancies.

## Figures and Tables

**Table 1:** Overview of current research involving GA-DM as a therapeutic agent in malignant and inflammatory diseases.

Cell Types	Disease to emulate	Anti-proliferative	Anti-metastatic	Source
PC-3 and LnCaP	Prostate cancer	yes	yes	Johnson et al. 2014
HT-144, 1359-mel, DM-331, J3, B16	Melanoma	yes	yes	Hossain et al. 2012
BCF-7. MDA-MB-231	Breast cancer	yes	Data not available	Wu et al. 2012
Ovx model, Raw 264 cell D-clone	Osteoporosis	yes	-	Miyamoto et al. 2009
T-Rex293 (transfected with pcDNA3-Aβ42-EGFP plasmid), T-Rex293-LEA15	Alzheimer’s disease	yes	-	Chakrabortee et al. 2012
IOMM-Lee, CH157MN	Meningioma	yes	yes	Das et al. 2015

## References

[1] Lindequist U, Niedermeyer TH, Julich WD (2005). The pharmacological potential of mushrooms. Evid Based Complement Alternat Med.

[2] Wasser SP (2011). Current findings, future trends, and unsolved problems in studies of medicinal mushrooms. Appl Microbiol Biotechnol.

[3] Wu GS, Lu JJ, Guo JJ, Li YB, Tan W (2012). Ganoderic acid DM, a natural triterpenoid, induces DNA damage, G1 cell cycle arrest and apoptosis in human breast cancer cells. Fitoterapia.

[4] Radwan FF, Perez JM, Haque A (2011). Apoptotic and Immune Restoration Effects of Ganoderic Acids Define a New Prospective for Complementary Treatment of Cancer. J Clin Cell Immunol.

[5] Bishop KS, Kao CH, Xu Y, Glucina MP, Paterson RR (2015). From 2000 years of Ganoderma lucidum to recent developments in nutraceuticals. Phytochemistry.

[6] Wachtel-Galor S, Yuen J, Buswell JA, Benzie IFF (2011). Ganoderma lucidum (Lingzhi or Reishi): A Medicinal.

[7] Radwan FF, Hossain A, God JM, Leaphart N, Elvington M (2015). Reduction of myeloid-derived suppressor cells and lymphoma growth by a natural triterpenoid. J Cell Biochem.

[8] Huang SZ, Ma QY, Kong FD, Guo ZK, Cai CH (2017). Lanostane-type triterpenoids from the fruiting body of Ganoderma calidophilum. Phytochemistry.

[9] Wei JC, Wang AH, Wei YL, Huo XK, Tian X (2017). Chemical characteristics of the fungus Ganoderma lucidum and their inhibitory effects on acetylcholinesterase. J Asian Nat Prod Res.

[10] Liu J, Shimizu K, Tanaka A, Shinobu W, Ohnuki K (2012). Target proteins of ganoderic acid DM provides clues to various pharmacological mechanisms. Sci Rep.

[11] Shi L, Ren A, Mu D, Zhao M (2010). Current progress in the study on biosynthesis and regulation of ganoderic acids. Appl Microbiol Biotechnol.

[12] Akihisa T, Nakamura Y, Tagata M, Tokuda H, Yasukawa K (2007). Anti-inflammatory and anti-tumor-promoting effects of triterpene acids and sterols from the fungus Ganoderma lucidum. Chem Biodivers.

[13] Xia Q, Zhang H, Sun X, Zhao H, Wu L (2014). A comprehensive review of the structure elucidation and biological activity of triterpenoids from Ganoderma spp. Molecules.

[14] Ruan W, Wei Y, Popovich DG (2015). Distinct Responses of Cytotoxic Ganoderma lucidum Triterpenoids in Human Carcinoma Cells. Phytother Res.

[15] Hossain A, Radwan FF, Doonan BP, God JM, Zhang L (2012). A possible cross-talk between autophagy and apoptosis in generating an immune response in melanoma. Apoptosis.

[16] Johnson BM, Doonan BP, Radwan FF, Haque A (2010). Ganoderic Acid DM: An Alternative Agent for the Treatment of Advanced Prostate Cancer. Open Prost Cancer J.

[17] Das A, Miller R, Lee P, Holden CA, Lindhorst SM (2015). A novel component from citrus, ginger, and mushroom family exhibits antitumor activity on human meningioma cells through suppressing the Wnt/beta-catenin signaling pathway. Tumour Biol.

[18] Galsky MD, Vogelzang NJ (2010). Docetaxel-based combination therapy for castration-resistant prostate cancer. Ann Oncol.

[19] Liu J, Shiono J, Shimizu K, Kukita A, Kukita T (2009). Ganoderic acid DM: anti-androgenic osteoclastogenesis inhibitor. Bioorg Med Chem Lett.

[20] Halaris AE, Belendiuk KT, Freedman DX (1975). Antidepressant drugs affect dopamine uptake. Biochem Pharmacol.

[21] Sotgia F, Fiorillo M, Lisanti MP (2017). Mitochondrial markers predict recurrence, metastasis and tamoxifen-resistance in breast cancer patients: Early detection of treatment failure with companion diagnostics. Oncotarget.

[22] Shafei A, El-Bakly W, Sobhy A, Wagdy O, Reda A (2017). A review on the efficacy and toxicity of different doxorubicin nanoparticles for targeted therapy in metastatic breast cancer. Biomed Pharmacother.

[23] Das A, Miller R, Lee P, Holden CA, Lindhorst SM (2015). A novel component from citrus, ginger, and mushroom family exhibits antitumor activity on human meningioma cells through suppressing the Wnt/beta-catenin signaling pathway. Tumour Biol.

[24] Foon KA, Takeshita K, Zinzani PL (2012). Novel therapies for aggressive B-cell lymphoma. Adv Hematol.

[25] Sumanasuriya S, De Bono J (2017). Treatment of Advanced Prostate Cancer-A Review of Current Therapies and Future Promise. Cold Spring Harb Perspect Med.

[26] Rotow J, Bivona TG (2017). Understanding and targeting resistance mechanisms in NSCLC. Nat Rev Cancer.

[27] Prayongrat A, Xu C, Li H, Lin SH (2017). Clinical outcomes of intensity modulated proton therapy and concurrent chemotherapy in esophageal carcinoma: a single institutional experience. Adv Radiat Oncol.

[28] Sun Y, Peng ZL (2009). Programmed cell death and cancer. Postgrad Med J.

[29] Duprez L, Wirawan E, Vanden Berghe T, Vandenabeele P (2009). Major cell death pathways at a glance. Microbes Infect.

[30] Golstein P, Kroemer G (2007). Cell death by necrosis: towards a molecular definition. Trends Biochem Sci.

[31] Auberger P, Puissant A (2017). Autophagy, a key mechanism of oncogenesis and resistance in leukemia. Blood.

[32] Bhutia SK, Dash R, Das SK, Azab B, Su ZZ (2010). Mechanism of autophagy to apoptosis switch triggered in prostate cancer cells by antitumor cytokine melanoma differentiation-associated gene 7/interleukin-24. Cancer Res.

[33] Hoelzer D, Gökbuget N (2012). Chemoimmunotherapy in acute lymphoblastic leukemia. Blood Rev.

[34] Opat S, Hawkes EA (2017). Chemoimmunotherapy May Not Be Dead Yet in Chronic Lymphocytic Leukemia, But Fludarabine Plus Cyclophosphamide Plus Rituximab Is Potentially Facing Life Support. J Clin Oncol.

[35] Reddy NM, Thieblemont C (2017). Maintenance therapy following induction chemoimmunotherapy in patients with diffuse large B-cell lymphoma: current perspective. Ann Oncol.

[36] Sehn LH (2010). A decade of R-CHOP. Blood.

[37] Harjunpaa A, Junnikkala S, Meri S (2000). Rituximab (anti-CD20) therapy of B-cell lymphomas: direct complement killing is superior to cellular effector mechanisms. Scand J Immunol.

[38] Mossanen M, Krasnow RE, Nguyen PL, Trinh QD, Preston M (2017). Approach to the Patient with High-Risk Prostate Cancer. Urol Clin North Am.

[39] Woyen AV, Laczkó G, Høyer S, Hegyi L (2017). The Missing Link in the Diagnostic Pathway of Prostate Cancer. Pathol Oncol Res.

[40] Aoun F, Bourgi A, Ayoub E, El Rassy E, van Velthoven R (2017). Androgen deprivation therapy in the treatment of locally advanced, nonmetastatic prostate cancer: practical experience and a review of the clinical trial evidence. Ther Adv Urol.

[41] Rucci N, Angelucci A (2014). Prostate cancer and bone: the elective affinities. Biomed Res Int.

[42] Liu J, Kurashiki K, Shimizu K, Kondo R (2006). Structure-activity relationship for inhibition of 5alpha-reductase by triterpenoids isolated from Ganoderma lucidum. Bioorg Med Chem.

[43] Lee N, Barthel SR, Schatton T (2014). Melanoma stem cells and metastasis: mimicking hematopoietic cell trafficking?. Lab Invest.

[44] Haque MA, Li P, Jackson SK, Zarour HM, Hawes JW (2002). Absence of gamma-interferon-inducible lysosomal thiol reductase in melanomas disrupts T cell recognition of select immunodominant epitopes. J Exp Med.

[45] Hathaway-Schrader JD, Doonan BP, Hossain A, Radwan FFY, Zhang L (2017). Autophagy-dependent crosstalk between GILT and PAX-3 influences radiation sensitivity of human melanoma cells. J Cell Biochem.

[46] Jemal A, Bray F, Center MM, Ferlay J, Ward E (2011). Global cancer statistics. CA Cancer J Clin.

[47] Miyamoto I, Liu J, Shimizu K, Sato M, Kukita A (2009). Regulation of osteoclastogenesis by ganoderic acid DM isolated from Ganoderma lucidum. Eur J Pharmacol.

[48] Edwards MH, Dennison EM, Aihie Sayer A, Fielding R, Cooper C (2015). Osteoporosis and sarcopenia in older age. Bone.

[49] Chenbhanich J, Thongprayoon C, Atsawarungruangkit A, Phupitakphol T, Cheungpasitporn W (2017). Osteoporosis and bone mineral density in patients with Wilson’s disease: a systematic review and meta-analysis. Osteoporos Int.

[50] Saito T, Sterbenz JM, Malay S, Zhong L, MacEachern MP (2017). Effectiveness of anti-osteoporotic drugs to prevent secondary fragility fractures: systematic review and meta-analysis. Osteoporos Int.

[51] Takahashi N, Udagawa N, Suda T (1999). A new member of tumor necrosis factor ligand family, ODF/OPGL/TRANCE/RANKL, regulates osteoclast differentiation and function. Biochem Biophys Res Commun.

[52] Yagi M, Ninomiya K, Fujita N, Suzuki T, Iwasaki R (2007). Induction of DC-STAMP by alternative activation and downstream signaling mechanisms. J Bone Miner Res.

[53] Chakrabortee S, Liu Y, Zhnag L, Matthews HR, Zhang H (2012). Macromolecular and small-molecule modulation of intracellular Abeta42 aggregation and associated toxicity. Biochem J.

[54] Corriveau RA, Koroshetz WJ, Gladman JT, Jeon S, Babcock D (2017). Alzheimer’s Disease-Related Dementias Summit 2016: National research priorities. Neurology.

[55] Ragel BT, Jensen RL, Gillespie DL, Prescott SM, Couldwell WT (2005). Ubiquitous expression of cyclooxygenase-2 in meningiomas and decrease in cell growth following in vitro treatment with the inhibitor celecoxib: potential therapeutic application. J Neurosurg.

[56] Preusser M, Berghoff AS, Hottinger AF (2013). High-grade meningiomas: new avenues for drug treatment?. Curr Opin Neurol.

[57] Piscevic I, Villa A, Milićević M, Ilić R, Nikitović M (2015). The Influence of Adjuvant Radiotherapy in Atypical and Anaplastic Meningiomas: A Series of 88 Patients in a Single Institution. World Neurosurg.

